# Nitrogen-metabolism related genes in barley - haplotype diversity, linkage mapping and associations with malting and kernel quality parameters

**DOI:** 10.1186/1471-2156-14-77

**Published:** 2013-09-04

**Authors:** Inge E Matthies, Stephan Weise, Jutta Förster, Viktor Korzun, Nils Stein, Marion S Röder

**Affiliations:** 1 Leibniz Institute of Plant Genetics and Crop Plant Research (IPK), OT Gatersleben, Corrensstr. 3, 06466, Stadt Seeland, Germany; 2 Saaten-Union Biotec GmbH, Hovedisser Str. 92, 33818, Leopoldshöhe, Germany; 3 KWS LOCHOW GMBH, Ferdinand-von-Lochow-Str. 5, 29303, Bergen, Germany

## Abstract

**Background:**

Several studies report about intra-specific trait variation of nitrogen-metabolism related traits, such as N(itrogen)-use efficiency, protein content, N-storage and remobilization in barley and related grass species. The goal of this study was to assess the intra-specific genetic diversity present in primary N-metabolism genes of barley and to investigate the associations of the detected haplotype diversity with malting and kernel quality related traits.

**Results:**

Partial sequences of five genes related to N-metabolism in barley (*Hordeum vulgare* L.) were obtained, i.e. nitrate reductase 1, glutamine synthetase 2, ferredoxin-dependent glutamate synthase, aspartate aminotransferase and asparaginase. Two to five haplotypes in each gene were discovered in a set of 190 various varieties. The development of 33 SNP markers allowed the genotyping of all these barley varieties consisting of spring and winter types. Furthermore, these markers could be mapped in several doubled haploid populations. Cluster analysis based on haplotypes revealed a more uniform pattern of the spring barleys as compared to the winter barleys. Based on linear model approaches associations to several malting and kernel quality traits including soluble N and protein were identified.

**Conclusions:**

A study was conducted to investigate the presence of sequence variation of several genes related to the primary N-metabolism in barley. The detected diversity could be related to particular phenotypic traits. Specific differences between spring and winter barleys most likely reflect different breeding aims. The developed markers can be used as tool for further genetic studies and marker-assisted selection during breeding of barley.

## Background

Nitrogen is an essential macronutrient in higher plants. It is part of many organic molecules, such as amino acids, proteins, chlorophyll, nucleotides, coenzymes and secondary metabolites. Higher plants have the ability to assimilate inorganic nitrate from the soil by reducing it to ammonium which is subsequently incorporated in the amino acid metabolism in the glutamate synthase cycle [1]. Photorespiration leads, besides a loss of CO_2_ and energy also to a stoichiometric release of NH_3_. This NH_3_ must be re-assimilated if the plant is not to lose all its organic nitrogen [2,3]. For barley, being a C3-plant, a higher photorespiration activity can be assumed in comparison to C4-plants, such as maize. It was shown for dicotyledonous plants, that C4-plants have higher nitrogen use efficiency than C3-plants [4].

N-use efficiency has been linked to the agronomic performance of crop plants [5,6] and genotype specific differences were described in several crop species (reviewed in [7]). In order to elucidate the genetic basis of N-use efficiency QTL mapping was conducted in segregating populations for agronomic performance under different N-input regimes [8-11] as well as for physiological traits and enzyme activities [12,13]. Several studies point out the central role of glutamine synthetase [14-16]. QTL mapping of grain protein content [17], nitrogen storage and remobilization [18] and yield related traits under two various N-input regimes [19] are reported for barley. Several QTL controlling nitrogen stress tolerance were found in wild barley introgression lines [20]. Though grain and malt nitrogen and protein content are common traits evaluated in malting quality studies [21,22] little is known about the impact of primary N-metabolism genes on malting quality in barley. Differential expression of genes of amino acid metabolism and protein metabolic processes were connected to seed germination and malting quality [23-25].

The prerequisite for intra-specific trait variation is intra-specific genetic variability in the responsible structural or regulatory genes or their regulatory sequences in the respective pathway. So far, little is known about intra-specific variability of primary N-metabolism genes in barley or other related species [26]. Therefore one aspect of this research was to investigate the genetic diversity present in five representative genes of the primary N-metabolism in barley.

Nitrate reductase, also well known as NAR (EC 1.6.6.1 and 1.6.6.2) catalyzes the reduction of nitrate (NO_3_^-^) to nitrite (NO_2_^-^) and is a key enzyme of nitrate assimilation in higher plants. In barley the presence of at least two isozymes of nitrate reductase [27] and the mapping of two loci *nar1* and *nar2* were reported [28,29].

Glutamine synthetase (GS) (EC 6.3.1.2), also called glutamate-ammonia ligase, plays an essential role in the metabolism of nitrogen by catalyzing the ATP-dependent condensation of glutamate and ammonia to form glutamine. Generally two types of glutamine synthetase occur in higher plants, a cytosolic isoform *GS1* and a plastid isoform *GS2*[16]. In wheat, ten GS sequences were classified which are differentially expressed in various tissues and appear to be developmentally regulated [30]. In higher plants, the assimilation of ammonia occurs primarily via the glutamate-synthase cycle which is catalyzed by glutamine synthetase and glutamate synthase. Ferredoxin-dependent glutamate synthase (GOGAT) (EC 1.4.7.1) is a flavoprotein catalyzing the formation of 2 mols of glutamate from 1 mol of glutamine and 1 mol of 2-oxoglutarate with ferredoxin as electron donor. GOGAT is not only involved in primary ammonium assimilation, but also in the reassimilation of ammonium released by photorespiration [2].

Aspartate aminotransferase (AAT), also called aspartate transaminase is a pyridoxal phosphate (PLP)-dependent transaminase enzyme (EC 2.6.1.1) and catalyzes the interconversion of aspartate and α-ketoglutarate to oxalacetate and glutamate. The reversible amino group transfer between aspartate and glutamate catalyzed by this enzyme is crucial in both amino acid degradation and biosynthesis.

Asparaginase (ASP) (EC 3.5.1.1) catalyzes the hydrolysis of asparagine to aspartic acid.

Many of the considered genes are known to be present in more than one copy or even gene families. For nitrate reductase in barley two isozymes and two genes were described [29,31]. Also for asparaginase in the *Arabidopsis* genome two genes *ASPGA1* and *ASPGB1* were found [32] and three genes for aspartate aminotransferase were described in rice [33]. While two genes encoding ferredoxin-dependent glutamate synthase with differential expression patterns are present in *Arabidopsis*[34], in wheat ten differentially expressed glutamine synthetase sequences were classified [30].

In the past years associations have emerged as powerful method to link molecular markers with phenotypic traits in many plant species such as barley [35]. In barley, association studies were conducted based on candidate gene approaches [36-44] as well as on the whole genome level [45-52]. Besides the primary assessment of haplotype diversity present in the N-metabolism candidate genes, we were interested to study the impact of these genes on malting quality related traits in barley such as soluble nitrogen and soluble protein. Phenotypic data derived from a database [53] were used. Furthermore, all five investigated genes were mapped in existing linkage maps of various barley mapping populations and the locations compared to known reported QTL.

## Methods

### Plant material and DNA isolation

The investigated plant material comprised 94 spring and 96 winter barley cultivars which were genotyped and used for association analysis. All 190 cultivars were of European origin characterized by specific malting and feeding qualities (Additional file 1). Seed samples were obtained from various breeders. Leaves were harvested from five to six seedlings and bulk genomic DNA was extracted according to a modified protocol of [54].

### Primer design, resequencing and SNP identification

The NCBI-database (http://www.ncbi.nlm.nih.gov/gene/) was used to retrieve mRNA and cds-sequences of the investigated genes in barley (Additional file 2). These were aligned using Sequencher™ vers. 4.06 software (http://www.genecodes.com) and the resulting consensus sequences formed the basis of PCR primer design using Primer3 software [55] by targeting amplicons in the size range of 500 to 800 bp (Additional file 3). The PCR protocol was modified from that described by [56]. The resulting PCR products were purified using a MinElute™ UF PCR purification kit (QIAGEN, Hilden, Germany) according to the manufacturer’s instructions. Amplicon sequencing was performed in a cycle sequencing reaction from both ends using the same primers as used for the PCR amplification.

Resequencing was performed with 16 diverse barley lines, including the winter varieties ‘Tiffany’, ‘Vanessa’, ‘Lomerit’, ‘Verena’, ‘Igri’ and ‘Franka’, the spring varieties ‘Brenda’, ‘Steina’, ‘Alexis’, ‘Steffi’, ‘Marthe’, ‘Steptoe’ and ‘Morex’, the parents of the Oregon Wolf Barley mapping population ‘OWB-dom’ and ‘OWB-rec’ [57] and a *Hordeum vulgare* ssp. *spontaneum* line HS584 described in [58]. The acquired sequences for each gene or fragment were aligned using the Sequencher^TM^ vers. 4.06 software and analyzed for SNPs. The assignment of exonic and intronic regions of a gene were determined by a spliced alignment between the genomic sequence and an EST or mRNA sequence using the tool Spidey (http://www.ncbi.nlm.nih.gov/spidey/).

For all indicated candidate genes in a key word search in the database HarvEST: Barley 1.83 (assembly 35) (http://www.harvest-web.org/) multiple contigs were found.

### Marker development and SNP genotyping

All detected SNPs were subjected to conversion into applicable molecular markers. Pyrosequencing assays were developed using PSQ Assay design software vers. 1.0.6 provided by Biotage (Uppsala, Sweden). Pyrosequencing analysis was performed on a PSQ™ HS9A device as previously decribed [38]. Genotyping by pyrosequencing was performed with all 190 barley cultivars used for association analysis and on the barley mapping populations used for linkage mapping. Finally, biallelic information of 33 high-quality SNP-markers derived from the five N-candidate genes was applied to further linkage and association mapping studies. Haplotypes for the individual genes were constructed from the combinations of all SNPs of the respective gene which were detected in the 16 barley lines used for re-sequencing and for which Pyrosequencing assays had been developed.

### Genetic mapping and cluster analysis

The chromosomal locations of the investigated candidate genes were analysed by using three doubled haploid (DH) mapping populations. The genes NAR, GS2 and AAT were mapped in a framework of 94 DH-lines of the population Steptoe × Morex [59], GOGAT was mapped in a framework of 73 DH-lines of the Igri × Franka population [60,61] and ASP was mapped in a framework of 94 DH-lines of the cross Morex × Barke [62]. In each case, several polymorphic SNP-markers for one gene were mapped and co-located in the respective mapping populations. Linkage mapping was conducted with the software package JoinMap vers. 4.0 [63] applying the function of Kosambi [64] to reveal the extent of linkage between the framework and the new markers.

Cluster analysis was carried out based on the haplotypes described in Additional file 3 using the NTSYS (Numerical Taxonomy and Multivariate Analysis System) vers. 2.2 software package [65]. The haplotype data were converted into a binary dataset and the pairwise similarity coefficient according to Dice [66] was computed. Cluster analysis was performed with the Unweighted Pair Group Method with Arithmetic Average (UPGMA) algorithm.

### Association analysis and linkage disequilibrium

Phenotypic data were acquired from the MetaBrew database (http://metabrew.ipk-gatersleben.de, [53]), which contains malting and kernel trait related data from public sources collected between the years 1985 and 2005. An unpublished updated version of this database contains varieties released till 2007. The malting quality traits selected for the association mapping were raw protein in the malt [%], Brabender [HE], diastatic power [WK], final attenuation [%], malt extract [%], fermentable extract [%], colour [EBC], pH-value in malt, friability [%], viscosity [mPas], VZ45 saccharification [%], glassiness [%], soluble N [mg/100 g dry matter], malting quality index (MQI), glume fineness, kernel quality and assortment [%], kernel formation [1-9], marketable yield [dt/ha] and grain yield [dt/ha]. These data were published from various German state trials in different years, at different locations including variable sets of varieties per trial and year. Each trait was covered by 2–103 single entries per variety (Additional file 4). For data analysis outliers deviating more than 20% from the mean were discarded. Mean values for each trait/variety combination were calculated over all available single entries (Additional file 5). Only mean values based on at least 20 single entries out of the total varietal set were taken into account. Due to availability of phenotypic data only up to 185 varieties were used for association analysis of the individual traits (Additional file 5). The Pearson product moment correlations of the resulting mean values were calculated using the software SigmaPlot v 11.0. The presence of multiple significant correlations among the traits demonstrated the interdependence of the investigated characters (Additional file 6).

To eliminate spurious associations due to population structure or kinship, both a STRUCTURE v2.2 analysis [67,68] using the Bayesian clustering approach to create a Q-matrix and a kinship analysis using the software SPAGeDi [69,70] were carried out, based on allelic status at 22 SSR loci in the 190 varieties. More details about the population structure in this set of barley accessions were previously described for a subset of 183 barley varieties which were used for association analysis [52]. The varieties were grouped into five subgroups corresponding to two groups of spring barleys and three groups of winter barley [52].

For association analysis three models were calculated using the software TASSEL vers. 2.1 [71]. These included a general linear model (GLM) taking eigenvalues into account (GLM-PCA), a GLM involving population structure with the assumption of five groups (GLM-Q5) and a mixed linear model including population structure and kinship (MLM-Q5 + K) [72,73]. For the MLM, the EMMA option [74] was used. Rare alleles were excluded from the association and the minimum allele frequency (MAF) threshold was set to 5%.

Linkage disequilibrium (LD) of in total 33 SNPs detected in all five investigated candidate genes was calculated using TASSEL vers. 3.0.

## Results and discussion

### Assessment of haplotype diversity

#### Nitrate reductase 1 (NAR)

We investigated a sequence of about 995 bp (Additional file 7) with 100% homology to the reported nitrate reductase cds sequence (NCBI accession number X57844; 1,231 – 2,225 bp [75]). Functional pyrosequencing markers were developed for 11 out of 16 SNPs identified by resequencing of 16 diverse barley reference lines. These were used to genotype a set of 190 barley varieties. All SNP sites were located in the coding region of NAR. However, only one SNP (NAR_SNP8) led to an amino acid exchange from glycine to alanine. All eleven investigated SNPs grouped the set of barley varieties in four haplotypes of which NAR_H1 predominated in the spring barleys while NAR_H2 is mostly found in winter barleys (Additional files 8 and 1).

#### Glutamine synthetase 2 (GS2)

Three non-overlapping genomic sequences containing exonic and intronic regions of the glutamine synthetase gene 2, i.e. from 5′ to 3′ GS37 (613 bp), GS36 (824 bp) and GS34 (1366 bp) were obtained (Additional file 7). High homology of these three sequences was detected to the barley mRNA for glutamine synthase 2 (NCBI accession X53580.1 [76]) as well as to some other cDNA sequences of barley (NCBI accessions: AK360336.1, AK364289.1, X16000.1) and wheat (NCBI accessions: GB169686.1, GQ169687.1). Spliced alignment showed that the three fragments GS34, GS36 and GS37 covered in total eleven different exons of the GS2 gene (Additional file 9).

Though eleven SNPs and three INDELS were used for marker development and genotyping of the varieties, only two haplotypes of GS2 were discovered. The predominating haplotype GS2-_H1 was present in 174 out of 190 varieties, while haplotype GS2_H2 was observed only in twelve spring varieties and one winter variety (Additional files 8 and 1). In hexaploid wheat the presence of two, six and two haplotypes were reported for Ta*GS2*-A1, Ta*GS2*-B1 and Ta*GS2*-D1 which are presumably syntenic loci on chromosomes 2AL, 2BL and 2DL, respectively [16].

#### Glutamate synthase (GOGAT)

We have obtained a partial sequence of 853 bp (Additional file 7) with homology to the ferredoxin-dependent GOGAT (NCBI accession no.: S58774, [77]). It contains two exonic regions and one intron with five SNPs which are defining three haplotypes. While the spring barleys are almost monomorphic showing haplotype GOGAT_H1, both haplotypes GOGAT_H1 and GOGAT_H2 are found in the winter barleys. The third haplotype GOGAT_H3 is extremely rare while occurring only twice (Additional files 8 and 1).

#### Aspartate aminotransferase (AAT)

We investigated a sequence stretch of 528 bp at the 3′-end of the AAT gene in barley consisting of 404 bp of coding sequence and after the stop codon of 124 bp of 3′-UTR (untranslated region) (Additional file 7). A homology of 100% was found in a BLAST search to *Hordeum vulgare* IDI4 mRNA for putative aspartate aminotransferase (NCBI accession no. AB206815, [78]). A total of four SNPs were discovered in the exonic region of which only one SNP (AAT_SNP4) led to an amino acid exchange from valine to isoleucine. All SNPs describe five haplotypes in the investigated set of barley varieties. The most frequent haplotype AAT_H1 was observed in 92 out of 95 spring barleys and in 35 out of 95 winter barleys, while haplotypes AAT_H4 and AAT_H5 represented by five and four varieties, respectively, were quite rare (Additional files 8 and 1).

#### Asparaginase (ASP)

We have investigated a partial sequence of 464 bp (Additional file 7) with homology to barley asparaginase mRNA (NCBI accession numbers: AF308474.1, AK363408 and AK368524, [79]) as well as to wheat cDNA (AK332789, [80]). In the exonic sequence part two SNPs were discovered which did not cause an amino acid exchange in the predicted protein sequence. Of the three haplotypes ASP_H3 occurred only once. ASP_H2 was found mostly in the spring barleys. ASP_H1 was the predominating haplotype in the winter barleys but occurred also in the spring barley gene pool (Additional files 8 and 1).

The observed numbers of haplotypes of two to five based on single SNPs in partial gene sequences are in the same range of magnitude like for other investigated genes in barley, such as β-amylase with six haplotypes [41], α-amylase with four haplotypes [38], sucrose synthase I with four haplotypes and sucrose phosphate synthase II with six haplotypes [40]. Also the predominance of certain haplotypes for either the spring or winter barley gene pool was found for other investigated genes [38,41].

### Cluster analysis based on haplotypes and linkage disequilibrium

**Figure 1:**
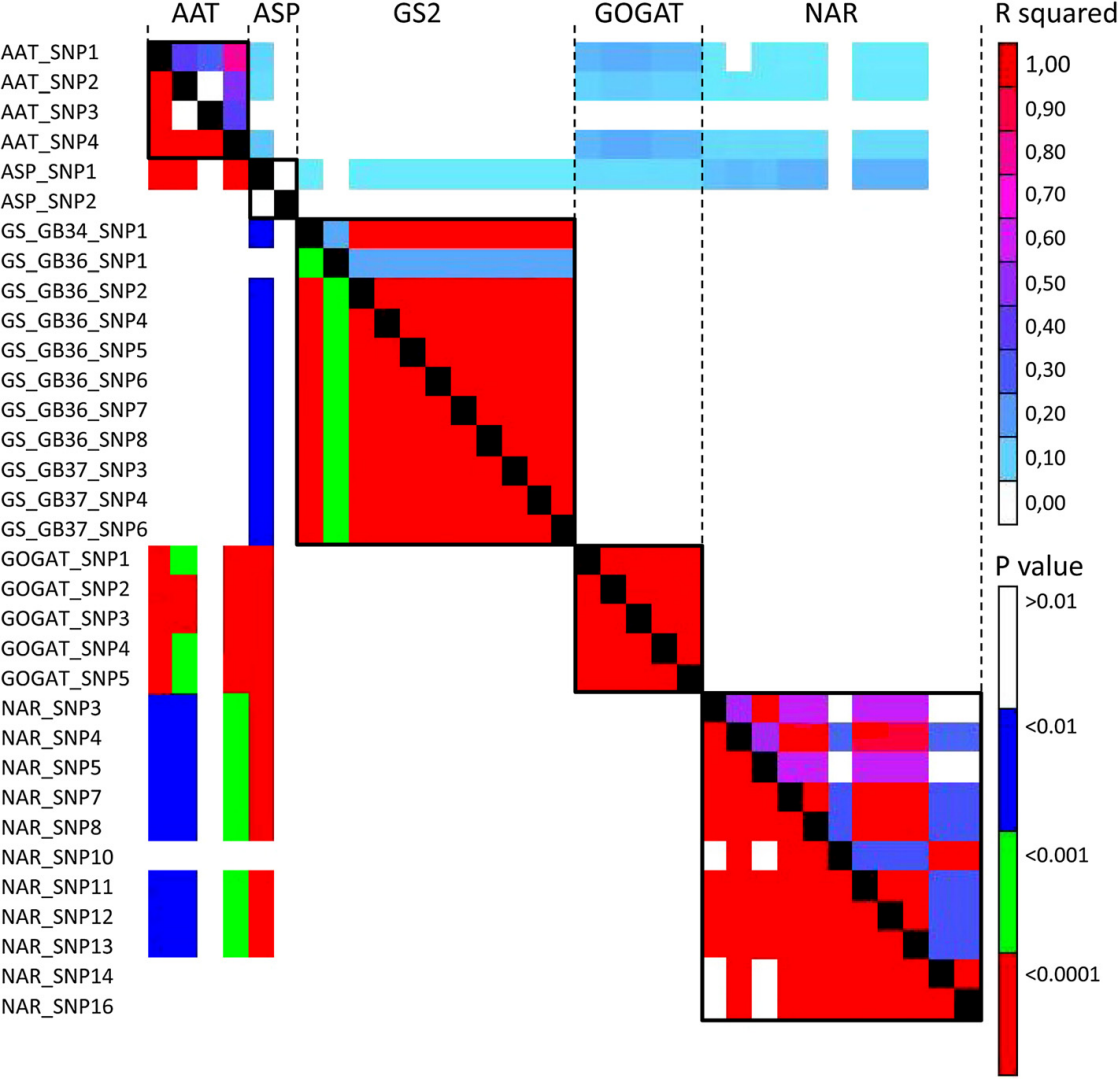
**Intragenic and intergenic linkage disequilibrium of five N-metabolism related genes based on 33 SNPs.** Depicted are the R^2^-values in the upper triangle and the P-values in the lower triangle. AAT = aspartate aminotransferase, ASP = asparaginase, GS2 = glutamine synthetase 2, GOGAT = glutamate synthase, NAR = nitrate reductase.

High intragenic LD among the individual 33 SNP markers was detected for all candidate genes except asparaginase (Figure 1), while the intergenic LD was absent or very low based on r^2^. High intragenic LD had been described in the past for other candidate genes in barley, such as β-amylase [41], α-amylase [38] and flowering time genes [43].

**Figure 2:**
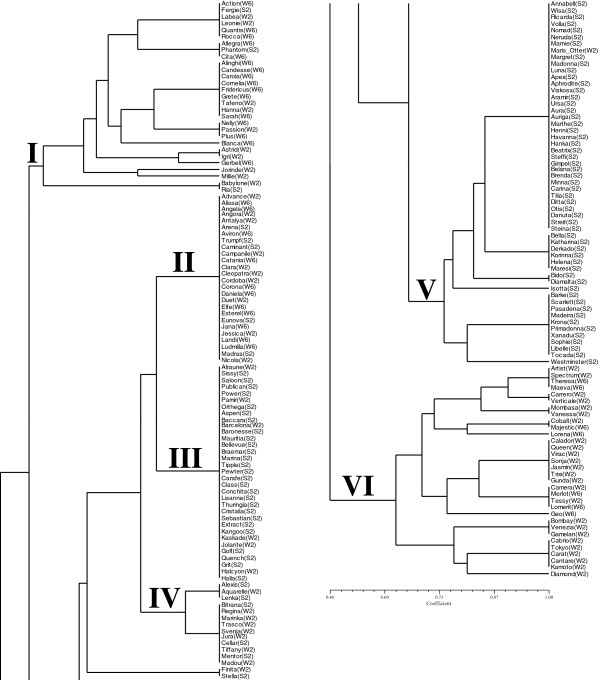
**Cluster analysis of 190 barley varieties based on haplotypes of five N-metabolism related genes.** S = spring type, W = winter type, 2 = two-rowed barley, 6 = six-rowed barley.

The cluster analysis based on the haplotypes of the five investigated N-metabolism genes led to 29 different combinations or meta-haplotypes (Figure 2). While spring barleys were only represented in 14 of the meta-haplotypes, winter barley was found in 27 meta-haplotypes. The winter barleys clustered mainly in group I and group VI, while groups II and IV were mixed of winter and spring genotypes and groups III and V consisted mainly of spring genotypes. While the groups containing the winter genotypes were separated in many small subgroups, group III was monomorphic consisting of 27 spring types and six two-rowed winter types. Group V contained also a large monomorphic subgroup consisting of 33 spring varieties and two winter varieties. The monomorphic group II consisted of winter and spring varieties. Overall, a larger average genetic distance was observed in the winter barley pool as compared to the spring barley gene pool (Table 1) based on analysis of haplotypes and analysis of single SNPs. The divergent patterns of the spring and winter varieties may be explained by different breeding aims for the two gene pools. The two-rowed spring barley varieties in this study were malting barleys for which low protein content in the grain is wanted. On the other hand most of the winter barley varieties are feeding types bred for high protein content in the grain. Only recently efforts were started to incorporate traits for malting quality in some two-rowed winter barley varieties. Therefore the breeding aims for winter barleys concerning the protein content were different in the past than those for the spring barleys as reflected in the phenetic tree. Prominent representatives for recent winter barley varieties with good malting quality properties are ‘Aquarelle’, ‘Tiffany’, ‘Madou’, ‘Regina’. These two-rowed accessions clustered in haplogroup IV.

**Table 1:** Average distances based on UPGMA (Unweighted Pair Group Method with Arithmetic Mean) cladogram option in TASSEL v. 3.0

**Average distance based on**	**190 varieties (winter and spring)**	**94 spring varieties**	**96 winter varieties**
33 SNPs in 5 genes	0.307	0.205	0.302
5 haplotypes of 5 genes	0.253	0.169	0.241

The obtained results may serve as base for selecting distant barley varieties for constructing mapping populations, and further genetic analysis of N-metabolism related genes regarding influencing N-efficiency during plant growth and malting properties.

### Linkage mapping and comparison to known QTL

**Figure 3:**
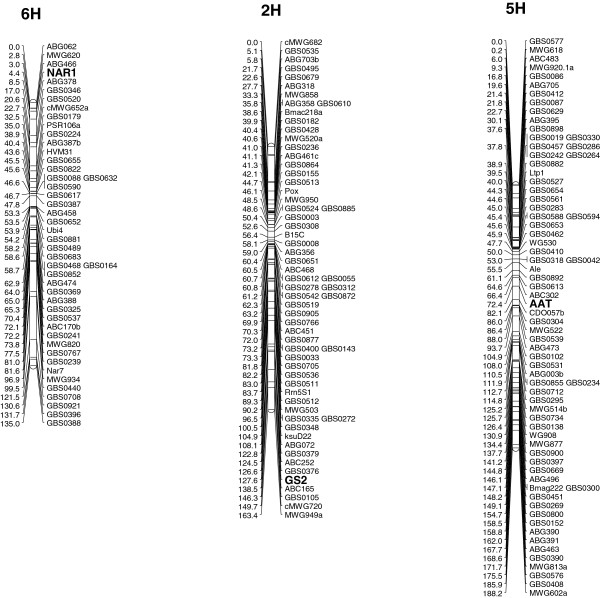
Genetic mapping of the genes nitrate reductase (NAR), glutamine synthetase 2 (GS2) and aspartate aminotransferase (AAT) in a chromosomal framework of the mapping population Steptoe × Morex.

The SNPs 11, 13, 14 found in NAR mapped on chromosome arm 6HS between RFLP-markers ABG466 and ABG378 (Figure 3). These results are in agreement with a report [29] which located the NADH-nitrate reductase structural gene *nar1* to barley chromosome 6H by the use of wheat barley addition lines. The RFLP marker ABG378B was reported as a marker for the *nar8* locus in barley (GrainGenes 2.0 database, http://wheat.pw.usda.gov/GG2/index.shtml). The *nar1* locus was reported in the same marker interval, while for various *nar7* loci different locations on 6H were reported including a location on 6HL. NAR loci were also found on other chromosomes, such as *nar2* and *nar5* on chromosomal bin 5H-BIN6; *nar4* and *nar6* on chromosomal bin 2H-BIN13 and *nar3* on chromosomal bin 7H-BIN5. While several studies report QTL for grain protein content and other N-metabolism related traits for marker Hvm74 on chromosome arm 6HL [17-19], this location does not coincide with the mapping of *nar1* on 6HS. Several QTL regions for N-metabolism related traits were reported on 6HS [18], however, a direct comparison with our mapping is difficult due to the use of different genetic markers and mapping populations. Also the QTL for grain protein content linked to marker ABG387 on 6HS [81] is located more proximal than the map location of *nar1*.

**Figure 4:**
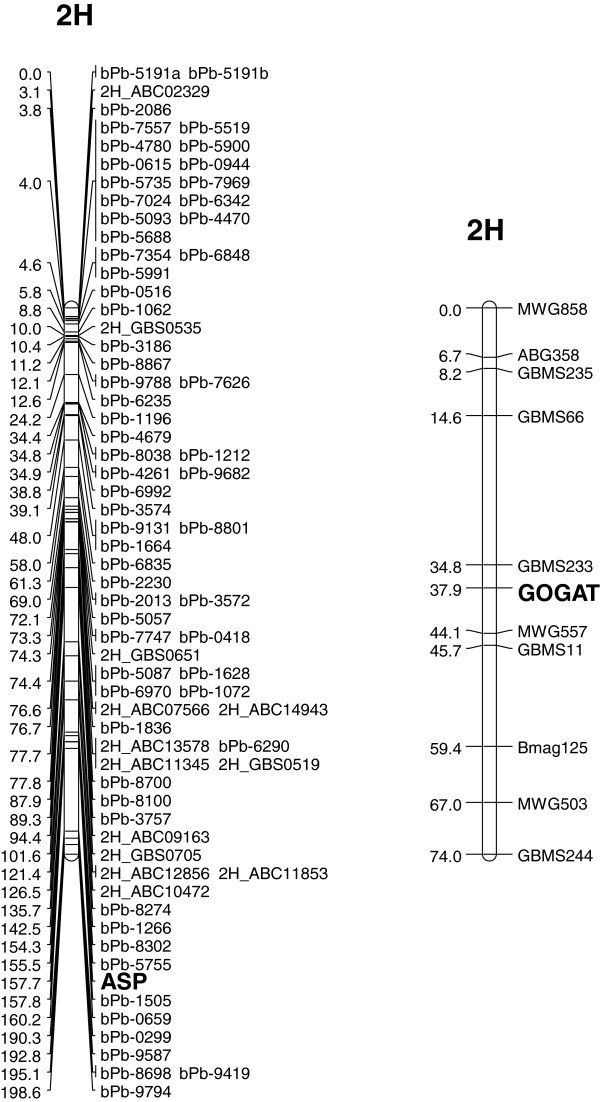
Genetic mapping of the gene asparaginase (ASP) in a chromosomal framework of the mapping population Morex × Barke and the gene glutamate synthase (GOGAT) in the mapping population Igri × Franka.

The linkage mapping of GS2 was conducted with various SNPs derived from all three examined sequences GB34, GB36 and GB37. Altogether, they co-localized on the long arm of chromosome 2HL between the markers GBS0376 and ABC165 (Figure 3), which is in agreement to the reported mapping of GS2 on group 2 chromosomes in wheat [10,16]. The detected SNPs of GOGAT were located on chromosome arm 2HS in between MWG557 and GBMS233 (Figure 4) by linkage mapping. This is in agreement with the chromosomal location based on wheat-barley telosomic addition lines [77].

A marker × nitrogen specific QTL for plant height was reported linked to marker EBMAC415 [19], which is located in the interval of GBM1016 and GBM1047. In the combined barley linkage map [59] the GBM1016 – GBM1047 interval encompasses the GBS0376 - ABC165 interval where GS2 was mapped. Two QTL for grain protein content were reported linked to markers ABG459 and Adh8 on chromosome arm 2HS [82]. The GOGAT mapped close to marker MWG557 which is reported more proximal in the consensus maps of the GrainGenes 2.0 database (http://wheat.pw.usda.gov/GG2/index.shtml). Also the grain protein QTL linked to MWG655e on 2HS [83] is located distal from the mapping of the GOGAT. A QTL for C-content in straw with specified N-fertilization coincided with the mapping of *Hv*-GOGAT [20].

The markers AAT_SNP2,3,4 mapped on the long arm of chromosome 5H between the RFLP-markers ABC302 and CDO57b; the next flanking SNP-markers were GBS0613 and GBS0304 (Figure 3). A QTL for grain protein content was reported on chromosome 5H (old nomenclature: chromosome 7) linked to marker ABC302 which is located in close proximity to AAT [83]. N-metabolism relevant QTL were reported for chromosome 5H [18]. Like for NAR, a comparison of the maps is difficult.

Linkage mapping placed the asparaginase on chromosome 2H between the DArT markers bPb-5755 and bPb-1505 (Figure 4). A linkage of marker bPb-5755 with a QTL for malt extract was reported [84].

The coincidence of a mapped marker of a candidate gene to a QTL is not a proof that there is a functional link between both of them. However, the knowledge of the mapping positions of important N-metabolism genes provides first evidences to study further their impact on relevant QTL.

### Associations to malting and kernel quality related traits

Previous studies have demonstrated the influence of the chosen statistical model or type of markers employed on the results and significance of marker-trait associations [50,52,85]. Here, three different statistical models were used to assess marker-trait associations between the N-metabolism candidate genes with malting and kernel quality traits (Table 2). Similar results were in most cases observed for a general linear model taking population structure into account (GLM – Q5) and a mixed linear model with population structure and kinship (MLM – Q5 + K), while a general linear model with eigenvalues (GLM – PCA) resulted in differing significant associations in many cases.

**Table 2:** Marker-trait associations in a set of 190 barley cultivars based on three statistical models

			**GLM - PCA**		**MLM - Q5 + K**		**GLM - Q5**	
**Fragment**	**SNP-/ID-marker**	**Trait**	**P**	**Sign.**	**P**	**Sign.**	**P**	**Sign.**
**Nitrate Reduktase 1 (NAR)**								
GB015-25	SNP3	Raw protein in malt [%]	0,0025	**	0.005	**	0.005	**
	SNP5	Raw protein in malt [%]	0,0025	**	0.005	**	0.005	**
	SNP11	Raw protein in malt [%]	0,002	**				
	SNP12	Raw protein in malt [%]	0,0054	**				
	SNP13	Raw protein in malt [%]	0,0053	**				
	SNP3	Soluble N [mg/100g_MTrS]	0,0126	**				
	SNP5	Soluble N [mg/100g_MTrS]	0,0126	**				
	SNP7	Fermentable Extract [%]	0,0067	**				
	SNP8	Fermentable Extract [%]	0,0067	**				
	SNP4	pH in malt					0.001	***
	SNP11	pH in malt					0.008	**
	SNP12	pH in malt					0.009	**
	SNP13	pH in malt					0.009	**
**Ferredoxin-dependent Glutamate Synthase (GOGAT)**								
GB005	SNP1	Marketable Yield [dt/ha]	0,0079	**	0.009	**	0.007	**
	SNP2	Marketable Yield [dt/ha]	0,0086	**	0.010	**	0.009	**
	SNP3	Marketable Yield [dt/ha]	0,0086	**	0.010	**	0.009	**
	SNP4	Marketable Yield [dt/ha]	0,0079	**	0.009	**	0.007	**
	SNP5	Marketable Yield [dt/ha]	0,0079	**	0.009	**	0.007	**
	SNP2	Final attenuation [%]			0.005	**	0.005	**
	SNP3	Final attenuation [%]			0.005	**	0.005	**
	SNP1	Viscosity [mPas]			0.000	***		
	SNP2	Viscosity [mPas]			0.000	***		
**Aspartate Aminotransferase (AAT)**								
GB056	SNP2	Fermentable Extract [%]			0.001	***	0.001	***
	SNP3	Fermentable Extract [%]	0,0041	**	0.002	**	0.003	**
	SNP2	Friability [%]			0.006	**	0.002	**
	SNP3	Malting quality index			0.014	**	0.011	**
	SNP3	pH in malt			0.006	**	0.002	**
	SNP4	pH in malt					0.007	**
**Asparaginase (ASP)**								
GB033	SNP1	Kernel formation [1-9]	0,0011	***				
	SNP1	Glume Fineness [1-9]	0,00043812	***				
	SNP1	pH in malt	0,00033973	***			0.005	**

GLM – PCA = General linear model considering Eigenvalues; MLM – Q5 + K = Mixed linear model considering population structure + kinship; GLM-Q5 = General linear model considering population structure assuming five groups. * P ≤ 0.05; **P ≤ 0.01; ***P ≤ 0.001.

Most of the associations were observed for nitrate reductase. NAR_SNP3 and NAR_SNP5 were significant for raw protein in malt with all three models. The same SNPs were significant for soluble N in the GLM-PCA model only. SNP11, SNP12 and SNP13 were found significant for raw protein in malt, and SNP7 and SNP 8 for fermentable extract in the GLM – PCA model, while four SNPs were significant for pH of the malt extract in malt with GLM - Q5.

All five SNPs of the GOGAT were significant for marketable yield with all three statistical models. The trait final attenuation was significant with SNP2 and SNP3 in two models, while significant associations with viscosity were only detected with the MLM – Q5 + K model.

Significant associations were detected for aspartate aminotransferase (ASP) ASP_SNP3 with all three models and for SNP2 with two models for the trait fermentable extract. Two models also gave significant associations for friability, malting quality index and pH in malt.

The marker GB033_SNP1 of asparaginase was associated with kernel formation, glume fineness and pH in the PCA-model. Though glutamine synthetase was reported as central enzyme of N-metabolism, no significant associations were detected for the tested markers of GS2.

The dependence of association results from the chosen statistical model is in agreement with other studies [86] and reflects different modes and degrees of compensation for population structure in the actual population used. Since our population was composed of spring and winter barleys, which represent quite divergent germplasm, the population was highly structured [52] and therefore the differences resulting from the statistical models used are not unexpected.

The occurrence of a marker-trait association is not a proof for a functional relationship. However, the associations for raw protein in malt and soluble N with nitrate reductase markers may point to an involvement of nitrate reductase in the expression of the respective traits.

## Conclusions

The presence of haplotype diversity in five genes related to the N-metabolism in barley is demonstrated. Different patterns for spring and winter barleys may reflect selection for diverse breeding aims during the process of variety development. Association studies indicated that the observed genotypic differences resulted in various phenotypic performance of certain malting and kernel quality traits. In total, 33 markers were developed allowing linkage and association mapping and haplotype analysis. These markers can be used for marker assisted selection during breeding and for further genetic analysis of N-metabolism related traits. Overall, the study encourages extended research of the genetic variability underlying N-metabolism related traits in barley. In barley recently novel genomic resources became available in form of ILLUMINA and INFINIUM chips for genome-wide SNP genotyping [62,87], as well as a draft sequence of the barley genome [88]. Nevertheless, the detailed analysis of genetic diversity of specific genes provides additional information which can help to elucidate the genetic components of traits and metabolic pathways.

## Authors’ contributions

IEM as the principal researcher conducted primer design, sequence analysis, marker development, statistical analysis, linkage and association studies. SW created the database for marker and traits and assisted in statistical analysis. JF and VK participated in genotyping. NS provided the mapping populations and data frameworks for linkage mapping. MSR coordinated the research, performed the cluster analysis, developed the concept and drafted the manuscript. All authors read and approved the final manuscript.

## Supplementary Material

Additional file 1 Haplotypes of candidate genes analysed in 190 barley varieties. Click here for file

Additional file 2 NCBI accession numbers of sequences used for contig formation and primer design. Click here for file

Additional file 3 Primer sequences used for (a) genomic resequencing and (b) for highthroughput genotyping by pyrosequening. Click here for file

Additional file 4 Trait statistics of single phenotypic values across 185 varieties (before elimination of outliers) for 22 traits. Click here for file

Additional file 5 Phenotypic data used for association analysis (mean values for each trait/variety combination after elimination of outliers). Click here for file

Additional file 6 **Correlations among traits.** * means P < 0.01. Click here for file

Additional file 7 List of investigated sequences including SNPs and INDELs. Click here for file

Additional file 8 SNP and haplotype frequencies of nitrate reductase gene, glutamine synthetase gene2, glutamate synthase gene, aspartate amonitransferase gene and asparaginase gene in 190 barley cultivars. Click here for file

Additional file 9 **Schematic representation of the investigated portion of gene glutamine synthetase 2 (GS2).** A spliced alignment was conducted between the partial cds sequence AK 360336.1 and the three investigated genomic sequences GS34, GS36 and GS37. Exonic regions are depicted as blocks. Numbers relate to the base pair units of the respective sequences. The three sequences GS37, GS36 and GS34 do not overlap and are in this order in 5′ → 3′ direction homologous to cds AK 360336.1. Click here for file
