# Supplementary material for: Nitrogen-metabolism related genes in barley - haplotype diversity, linkage mapping and associations with malting and kernel quality parameters
Source: BMC Genet. 2013 Sep 4;14:77. doi: 10.1186/1471-2156-14-77 (PMC3766251; doi:10.1186/1471-2156-14-77)
Supplement: Additional file 2 — NCBI accession numbers of sequences used for contig formation and primer design. [file 1471-2156-14-77-S2.pdf]

Additional file 2: NCBI accession numbers of sequences used for contig formation and primer design.

**Gene**	**NCBI accession**
Nitrate reductase	X57844; UniGene Hv. 20595
Glutamine synthetase	X1600; X53580; X69087; UniGene Hv. 331
Ferredoxin-dependent glutamate synthase	BJ478274; BJ551573; S58774
Aspartate aminotransferase	AB206815; AF017431; AF017432; Unigene Hv. 261777
Asparaginase	AAG28786; AF308474: UniGene Hv. 9579
