# Supplementary material for: Nitrogen-metabolism related genes in barley - haplotype diversity, linkage mapping and associations with malting and kernel quality parameters
Source: BMC Genet. 2013 Sep 4;14:77. doi: 10.1186/1471-2156-14-77 (PMC3766251; doi:10.1186/1471-2156-14-77)
Supplement: Additional file 7 — List of investigated sequences including SNPs and INDELs. [file 1471-2156-14-77-S7.docx]

**Additional file 7:** List of investigated sequences including SNPs and INDELs.

Intronic and UTR sequences are underlined;

Developed SNP and INDEL marker without predicted amino acid exchange are marked in blue, with predicted amino acid exchange in red;

Detected SNPs for which no genotyping marker was developed are marked in yellow.

**Nitrate reductase:**

>NAR_GB15,25

CTCGACATCCCGGACAAGCCCAACAAGTACGGCCGGTACTGGTGCTGGTG

CTTCTGGTCGGTAGAGATCGAGGTGCTGGACCTGCTGGGCGCCAAGGAGG

TGGCGGTGCGCGCGTGGGATCAGACCCACAACACCCAGCCGGAGAAGCTC

ATCTGGAACCTCATGGGCATGATGAACAACTGCTGGTTCAAGATCAAGGT

CAACGTGTGCCGCCCCCACAAGGGCGAGATCGGGCTGGTGTTCGAGCACC

CCACGCAGCCCGGGAACCAGACCGGCGGATGGATGGCGCGCCAGAAGCAC

CTCGAGACGGC(C/T)GAGGCGGCGGCGCCGGGGCTCAAGCGGAGCACGT

CCACGCCGTT(C/T)ATGAACACCGCCGGGGACAAGCAGTTCACCATGTC

CGAGGTGCGCAAGCACGGGTCCAAGGAGTCGGCGTGGATCGTGGTGCACG

G(C/T)CACGTCTACGACTGCAC(C/G)GCCTTCCTCAAGGACCACCCCG

GCGGCGCCGACAGCATCCTTATTAACGC(A/C)GGATCCGACTGCACCGA

GGAGTTCGACGCCATCCACTCCGACAAGGCCAAGGCGCTGCTCGACACCT

ACCGCATCGGCGAGCTGATCACCACGGGGACGGGGTACAACTC(C/G)GA

CAACTCGGTGCA(T/C)GG(A/T)GGGTCCAGCCTATCCCACCTCGCACC

GATCCGCGAGGCCACCAAGGTCGCCGGCGCGCCCATCGCGCTCTCCAGCC

C(G/A)CGCGAGAAGGTCCC(A/G)TG(T/C)CGCCTCGTCGACAAGAAG

GAGCTCTCCCACGACGTCCGCCTCTTCCG(C/T)TTCGCGCTGCCGTCCT

CCGACCAGGTGCTCGGCCT(C/G)CCCGT(C/T)GGCAAGCACATCTTCG

T(T/G)TGCGCCACCATCGACGGCAAGCTCTGCATGCGCGCCTACACGCC

GACGAGCATGGTGGACGAGATCGG(G/C)CAGTTCGAGCTGCTCGTCAAG

GTCTACTTCAGGGACGAGCACCCAAAGTTCCAATACGGCGGGCTCATGAC

GCAGTACCT

**Glutamine synthetase:**

>GS_GB034

ATGCCTGGACAGGTAAGCCTCCGTATTTATATGCGTGCATGTATGCTTGT

TATGTGTGAATATGTGTGATGATTGCGACCTCTTCTTTCTTTCAAAGGAA

AATCGAACTGTTTAATAAGTGAAAATTCACAACCTGTATTCATGCAGTGG

GAGTACCAGGTTGGACCCAGCGTTGGTATTGATGCAGGAGACCACATATG

GGCTTCCAGATACATTCTCGAGGTACTTCGAACAACTATCCATGGTTCCC

TGATGGCCTGATACCACAGAGCTTTTGTTATTTGCTTTTAGTTAGCTTAG

TATGGCTAATAAGTCAACTTTCTTTGTTACATTATCGTAAACCTGATAGG

TGCATGTGCTATGAGCTTATGATTTATTCTGACACTTTGAAGAATGCCAT

TCTTAAATACCATAAAAACAGTTTTCGCTAAAACATCAGAACCCATAAAT

CCATAACTGACCAGGTGACCATCCTTTGTGATATTAATTTTCTCATTAAT

AAGCATTTCTAATTAACATCCTACTCACTGTAATCAGAGAATCACGGAGC

AAGCTGGTGTGGTGCTCACCCTTGACCCAAAACCAATCCAGGTATATTTC

TGTAAGTTGTTTGATGAAGCAATTTATATATTAGAAGTTCGCAGTCTGAA

GATTTATCTGATGTAGGGTGACTGGAACGGAGCTGGCTGCCACACAAACT

ACAGGTTCCATTCTGTTATGTTAATATTTGTTCATCGTGCGTAACTTTTA

TAAAGTATATCTTGTCGTTTTCTTTTGAGAAACATATATCTTGCCGCATG

TATATATTGAAAAAAAGCATCAGAAAACCTATTGCAGCTGACTAGCTGTG

TTAGAGTAAATACTAAGGAAAAATATTCTT(T/C)GAAATGCAATGTTTC

AAACAGGGCATGGATGTTCAGCGCTGGTTATTTTAAAATTACTATAAATG

TTATTCCGAGTAGACATGTATTTACTATAGTTAGTTACTAACGGTTTAAG

TAGTTTTCTCATTACTCGATAGAGTATCACCTTAAATTGCAAGTGAAAAA

CTGTGTTTTCTTCTTGTGGTAAATGTAGCACATTGAGCATGCGCGAGGAT

GGAGGTTTCGACGTGATCAAGAAGGCAATCCTGAACCTTTCACTTCGCCA

TGACTTGCACATAGCCGCATATGGTGAAGGAAACGAGCGGAGGTTGACAG

GGCTACAYGAGACAGCTAGCATATCAGACTTCTCATGGGTATGGGTGGAG

CAAACCTTTTCTTTCTTTTTTATTTGTATTTTCCTTCCCTATTTATTTAC

AAGTTATACTGATCACAACACTTTGTTTATCAGGGTGTGGCGAACCGTGG

CTGCTCTATTCRTGTGGGGC

>GS_GB036

GGCATGTAGTGCCCTGAGTTCTTTTGCGGCAAGGGTCTATGCCRAGATTW

CCAGRGCAWGGKWTGYSTACTTTCTATTTCTGCTACTGACAAGAAAATGC

AATGCTACTGTTTCAGACGATTTCGAAGCCAGTGGAGGACCCGTCAGAGC

TGCCGAAATGGAACTACGACGGATCGAGCACGGGGCAGGCTCCTGGGGAA

GACAGTGAAGTCATCCTATAGTAAGGGGACAATTACAGTTCATGTGTTCT

TCAGCCTTGCACACACAGTCTAATAC(T/C)GTAATCTCGATTTCGACTT

T(T/G)GCTGACTCTGTTCATGTTTTTCTGTCCTGCTTTCAGCCCACAGG

CCATATTCAAGGACCCATTCCGAGGAGGCAACAACATACTGGTACCTTTC

TTCTGGATGTGCTTTA(C/T)GCTAATCATGAAGAAGTGATTAGTA[GTG]

CTAGTGGCCACTTTACTGGTTACA(T/C)(G/A)TATACAATTGGC(G/A)

TGTTA(G/C)TTAATACGGAATTTGTTTTTTCAGGTTATCTGTGACACCTA

CACACCACAGGGGGAACCCATCCCTACTAACAAACGCCACATGGCTGCACA

AATCTTCAGTGACCCCAAGGTCACTTCACAAGTGCCATGGTAACTA(C/G)

GCAT[C]GAGT(G/A)CC(T/C)GTGCTATATAAAATCCTGCTATTTTCTG

ATCTCGCTTTTGTTTGCATACGAAACTGAAACATTTGCTTTTCTAGGTTCG

GAATCGAACAGGAGTACACTCTGATGCAGAGGGATGTGAACTGGCCTCTTG

GCTGGCCTGTTGGAGGGTACCCTGGCCCCCAAGGATCACATCGAGAAGCTT

GATCTATCA

>GS_GB037

CTCTTTGGTTTTTGGCCTGGTTTGCCTCTGTTGCAGCTGCAAGGGGCGCC

TCAAATCTCGTCCACCCAGAGCCCTATCGCCTAATCAAGAGCCAGATGCC

ACTGCCCGCCCCGCGGCCACTAGAATCTCCCCCCGCAATCTAAAATACTT

ATACCACCCACCCCCCGCTTCCATAAATATTATTTCCAGCGCGATCTCAC

AGGTCGCCCCC(C/T)C(T/C)TCCCTCCCTC(G/T)CCTCGCCC(G/T)

C[G]TCGCC[C]GTCTCTCTGGTGAGCACCACGGAGTTCTCGATGCAATG

CTTCTTCGATTCTCTGTTGCATTCG/TCC/TGC/TC/TGC[C]G/AG/TT

CGATCGGCGGCAATGGCC[C]TGCTTG[CTTTG]CTTTGCT[TTGCT]GC

GACTGATCACGGGCTATCTCTCTTGCTGCAGGTTT(A/C)GGGGC(C/G)

(G/A)CGGAGTCGCTGTA(C/A)GTAAGTAAGTA(A/G)GTACGTAGAGA

CGACGATGGCGCAGGCGGT(T/G)GTGC(A/C)GGCGATGCAGTGCCAGG

TGGGGGTGAGGGGCAGGACGGCCGTCCCGGCGAGGCAGCCCGCGGGCAGG

GTGTGGGGCGTCAGGAGGGCCGCCCGCGCCACCTCCGGGTTCAAGGTGCT

GGCGCTCGGCCCGGAGACCACCGGGGTCATC

**Glutamate synthase:**

>GOGAT_GB005

CCGGTGTTGGATTTGTCGCAAACTTGAGTAATGAGCCTTCGTTCAACGTT

GTCCGTGATGCTCTTACAGCTCTTGGGTGCATGGAGCACCGTGGTGGCTG

TGGAGCCGACAACGACTCTGGTGACGGGGCAGGATTGATGAGCGGCATAC

CATGGGACTTGTTTGATGACTGGGCCAGCAAGGAAGGGCTTGCTCCTTTT

GAAAGAACACATACAGGTGTAGGCATGGTCTTCCTTCCACAAAACGAGAA

TTCCATGGAAGAAGCAAAAGCTGGTAATGATTCTGATGCTTTACCACAAT

ACG(C/T)CGCCCACATTGTGGATAATTTCTTGTCTTCCTTTTTTGCCGG

GATTTTGTCTTTA(A/G)AGTGCGACACTTCCTAGTTTTATATGG(T/C)

CCGAGATTTCTGCTTGGCGATGCATGTCCTAGTAAATCTGAGTCCGATCT

CTTCGGACTCGTAATGCTGCCATACTTTGGTTGCAATGCCATCGTGGTGC

TTGAAACCTATGCTTGGAAAGTT(T/C)TTTTGCAGGAAAAGTTTGTGCC

CTTGGTATAAGAACTTTCCAAAAGGAATTTATTTATTTTGTATTGACAGT

CAAATTACACATGTGATTTTTCAGCTCTACTGTA(T/C)CACAACACACA

CAGCTTACTAAGAGTCTATTCATTTTTGCAGCTGTTGAGAAGGTTTTTAC

AGATGAAGGCCTTGAGGTTCTTGGCTGGAGACCCGTTCCTTTCAATCTAT

CAGTGGTAGGCCGCAATGCAAAAGAAACAATGCCTAATATACTCCAGATA

TTTGTGAGAATTGCGAAAGAAGATGACGCTGATGACATAGAGAGAGAATT

ATACATCTGCGGGAAGCTGAAAA

**Aspartate aminotransferase:**

>GB_056_AAT

GTAAGGCAGGCACACTTCTTCCTCACATTCGCCACCTCCACGCCGATGCA

ATCAGCAGCAGCGGCGGCC(T/C)TGAGAGCACCGGACAGCTACTTTGAG

GAGCT(G/C)AAGAGGGACTACGGCGCAAAGAAAGCGCTGCTGGTGGACG

GGCTCAAGGCGGCGGGCTTCATCGTCTACCCTTCGAGCGG(G/A)ACCTA

CTTC(G/A)TCATGGTCGACCACACCCCGTTCGGGTTCGACAACGACGTC

GAGTTCTGCGAGTACTTGATCCGCGAGGTCGGCGTCGTGGCCATCCCGCC

GAGCGTGTTCTACCTGAACCCGGAGGACGGGAAGAACCTGGTGAGGTTCA

CCTTCTGCAAGGACGACGACACGCTGAGGGCGGCGGTGGACCGGATGAAG

GCCAAGCTCAGGAAGAAATGATTGAGGGGCRACATCTGTGTAGCCGTTGA

TGATTGGTTGGTGAATAAAGACCTGCTCGATCTGATCTGATCTGCATTCC

GGTGCGATGCATGTAACACTTTTTTCTAGCAGCAGTTATCTACTAAAAAA

AAAAAAAA

**Asparaginase:**

>ASP_GB033

TGCGCCKTGGTGGAMCTGAACGGCTTCACGGCGGCGGCCACCTCCACGGG

CGGGCTCATGAACAAGATGACGGGCCGCATCGGCGACTCCCCGCTCATCG

GCGCCGGCACCTACGCGTGCGGGCACTGCGCCGTGTCGTGCACGGGCGAG

GGAGAGGCCATCATCCGCTCTACGCTGGCACGGGACGT(G/C)GCGGCCG

TCATGGAGTACAAGGGCCTCCCGCTGCAGGAGGCCGTGGACTTCTGCGTC

AAGGAGCGGCTCGACGAGGGGTTCGCCGGGCTCATCGCCGTGTC(G/A)G

GCACCGGCGAGGTGGCATACGGGTTCAACTGCACCGGCATGTTCAGGGGC

TGCGCAACCGAGGACGGCTTCATGGAGGTCGGCATCTGGGAGTGAGCAAG

TGCGCGAGCTCAGCCGAGAGGATGTGTCCGCGCTATTGTGTGTACATACA

TTTGGCASWTCCTGTGATTGCC
