# Supplementary material for: Nitrogen-metabolism related genes in barley - haplotype diversity, linkage mapping and associations with malting and kernel quality parameters
Source: BMC Genet. 2013 Sep 4;14:77. doi: 10.1186/1471-2156-14-77 (PMC3766251; doi:10.1186/1471-2156-14-77)
Supplement: Additional file 9 — Schematic representation of the investigated portion of gene glutamine synthetase 2 (GS2). A spliced alignment was conducted between the partial cds sequence AK 360336.1 and the three investigated genomic sequences GS34, GS36 and GS37. Exonic regions are depicted as blocks. Numbers relate to the base pair units of the respective sequences. The three sequences GS37, GS36 and GS34 do not overlap and are in this order in 5′ → 3′ direction homologous to cds AK 360336.1. [file 1471-2156-14-77-S9.pdf]

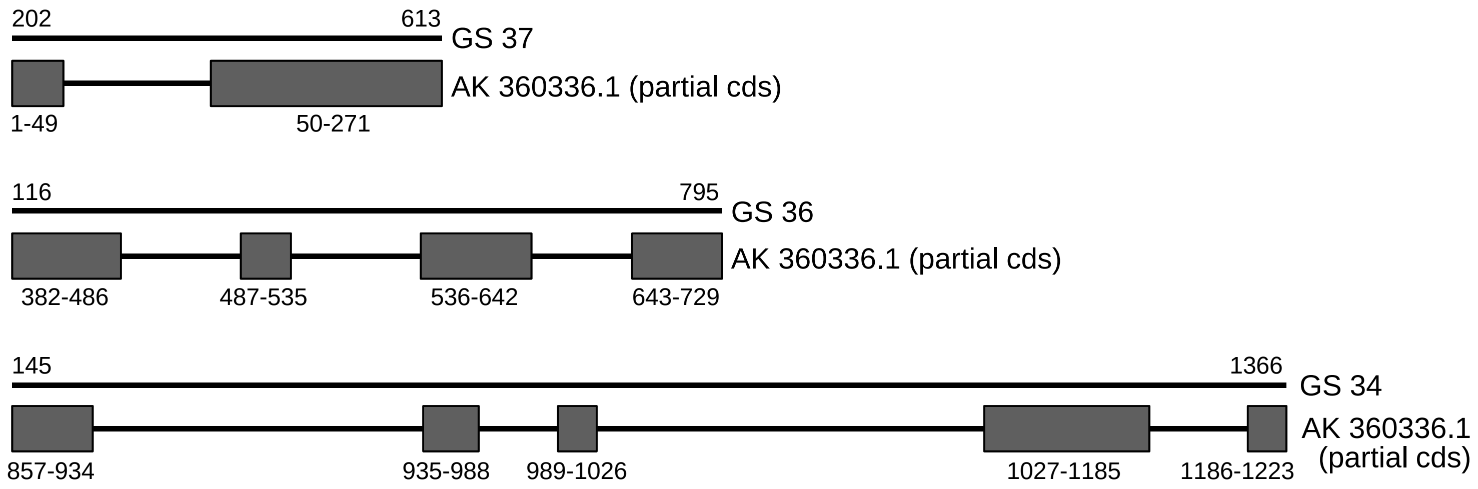

Additional file 9: Schematic representation of the investigated portion of gene glutamine synthetase 2 (GS2). A spliced alignment was conducted between the partial cds sequence AK 360336.1 and the three investigated genomic sequences GS34, GS36 and GS37. Exonic regions are depicted as blocks. Numbers relate to the base pair units of the respective sequences. The three sequences GS37, GS36 and GS34 do not overlap and are in this order in 5'→ 3' direction homologous to cds AK 360336.1.
